# Staged endovascular repair of post-dissection thoracoabdominal aortic aneurysm using false lumen stent grafts placement

**DOI:** 10.1093/jscr/rjaf336

**Published:** 2025-05-23

**Authors:** Hironobu Nishiori, Hideki Ueda, Michiko Watanabe, Hiroki Kohno, Kaoru Matsuura, Tomohiko Inui, Hiroki Ikeuchi, Tomoyoshi Kanda, Chihiro Ito, Hiroaki Yamamoto, Yusuke Shibata, Takashi Yamamoto, Maiko Nagahama, Goro Matsumiya

**Affiliations:** Department of Cardiovascular Surgery, Chiba University Hospital, 1-8-1, Inohana, Chuo-Ku, Chiba City, Chiba 260-8677, Japan; Department of Cardiology, Minami-Senju Hospital, Tokyo, Japan; Department of Cardiovascular Surgery, Chiba University Hospital, 1-8-1, Inohana, Chuo-Ku, Chiba City, Chiba 260-8677, Japan; Department of Cardiovascular Surgery, Chiba University Hospital, 1-8-1, Inohana, Chuo-Ku, Chiba City, Chiba 260-8677, Japan; Department of Cardiovascular Surgery, Chiba University Hospital, 1-8-1, Inohana, Chuo-Ku, Chiba City, Chiba 260-8677, Japan; Department of Cardiovascular Surgery, Chiba University Hospital, 1-8-1, Inohana, Chuo-Ku, Chiba City, Chiba 260-8677, Japan; Department of Cardiovascular Surgery, Chiba University Hospital, 1-8-1, Inohana, Chuo-Ku, Chiba City, Chiba 260-8677, Japan; Department of Cardiovascular Surgery, Chiba University Hospital, 1-8-1, Inohana, Chuo-Ku, Chiba City, Chiba 260-8677, Japan; Department of Cardiovascular Surgery, Chiba University Hospital, 1-8-1, Inohana, Chuo-Ku, Chiba City, Chiba 260-8677, Japan; Department of Cardiovascular Surgery, Chiba University Hospital, 1-8-1, Inohana, Chuo-Ku, Chiba City, Chiba 260-8677, Japan; Department of Cardiovascular Surgery, Chiba University Hospital, 1-8-1, Inohana, Chuo-Ku, Chiba City, Chiba 260-8677, Japan; Department of Cardiovascular Surgery, Chiba University Hospital, 1-8-1, Inohana, Chuo-Ku, Chiba City, Chiba 260-8677, Japan; Department of Cardiovascular Surgery, Chiba University Hospital, 1-8-1, Inohana, Chuo-Ku, Chiba City, Chiba 260-8677, Japan; Department of Cardiovascular Surgery, Chiba University Hospital, 1-8-1, Inohana, Chuo-Ku, Chiba City, Chiba 260-8677, Japan

**Keywords:** endovascular repair, false lumen stent graft, post-dissection aortic aneurysm

## Abstract

We report a false lumen stent graft technique to close the intimal tears at the visceral segment for post-dissection thoracoabdominal aneurysm after initial thoracic endovascular aortic repair. Following abdominal endovascular aortic repair, a stent graft was then deployed in the false lumen, and successfully closed the intimal tears at the visceral segment. The residual re-entries at the iliac level were closed 2 years after the prior endovascular aortic repair, once a collateral network for spinal cord perfusion had developed, minimizing the risk of spinal cord ischemia. This endovascular strategy successfully reduced a size of post-dissection thoracoabdominal aneurysm.

## Introduction

Chronic type B aortic dissection (CTBAD) often results in false lumen (FL) dilation due to residual intimal tears. Even after closure of the primary entry, inflow into the FL persists when the tear is located at the visceral segment, leading to aneurysmal enlargement. A previous study reported a case where FL stent graft placement successfully covered the intimal tears at the visceral segment as part of a staged repair for post-dissection thoracoabdominal aortic aneurysm (TAAA) [1]. This report presents the second stage of endovascular repair for completing the treatment of CTBAD with FL stent graft placement and results obtained with reduction of the TAAA without spinal cord ischemia (SCI).

## Case report

**Figure 1:**
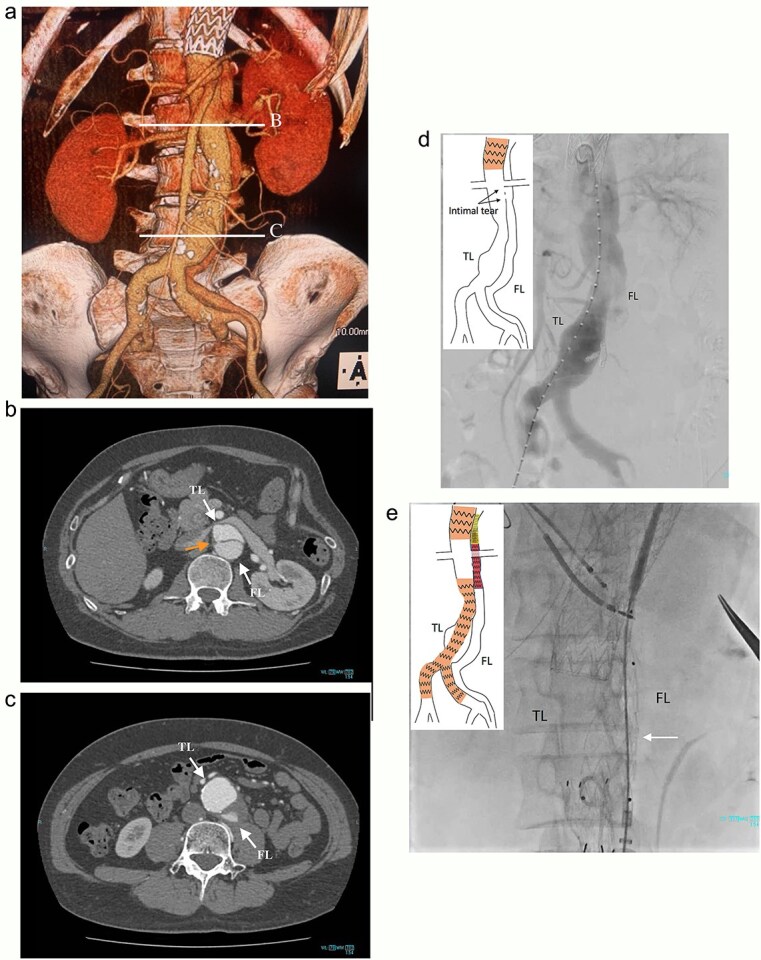
Preoperative CT 3D reconstruction showing the FL extended from the descending aorta to the external iliac artery (a), contrast-enhanced CT axial images at the renal artery level with an intimal tear (b; arrow), and the abdominal aortic aneurysm level (c), and the intraoperative angiography showing the FL were contrasted through intimal tear at the visceral segment (d), Endurant II iliac leg placed in the FL closing intimal tears near the renal artery (e; white arrow). TL: true lumen; FL: false lumen.

A 46-year-old man, with a history of noncompaction cardiomyopathy, underwent emergency zone 3 thoracic endovascular aortic repair (TEVAR) using TAG (WL Gore & Associates, Newark, DE, USA) for complicated type B acute aortic dissection. Postoperative contrast-enhanced computed tomography (CT) imaging showed the FL began from the descending aorta and extended into the left external iliac artery (EIA) (Fig. 1a), and several residual re-entries at the renal artery level (Fig. 1b) and one at the left EIA level. Both renal arteries were also originated from the true lumen. Both the FL and the post-dissection TAAA were dilated to diameters of 23 and 53 mm, respectively (Fig. 1c). Considering impaired cardiac function with the ejection fraction of 24%, the endovascular treatment was performed with intimal tear closure using FL stent graft placement as previous study reported [1]. First, endovascular aortic repair (EVAR) was completed with Excluder Aortic Extender (WL Gore & Associates, Newark, DE, USA) below the renal artery, followed by Excluder main body (WL Gore & Associates, Newark, DE, USA) landing on bilateral common iliac arteries. This excluded abdominal aortic aneurysm. Second, we placed a 10 × 40 mm Epic vascular self-expanding stent (Boston Scientific, Marlborough, MA, USA) in the FL at the level of distal end of the TAG. Then, the intimal tears at the renal artery level were closed using Endurant II iliac leg (cat No. ELW1624C94 Medtronic, Dublin, Ireland) from the FL side, overlapping with Epic stent proximally, and Aortic Extender distally (Fig. 1d and e). The retrograde angiography from left femoral artery sheath showed residual blood flow blowing up into the FL from the re-entry at the left EIA, and some lumbar arteries originating from the FL were contrasted.

**Figure 2:**
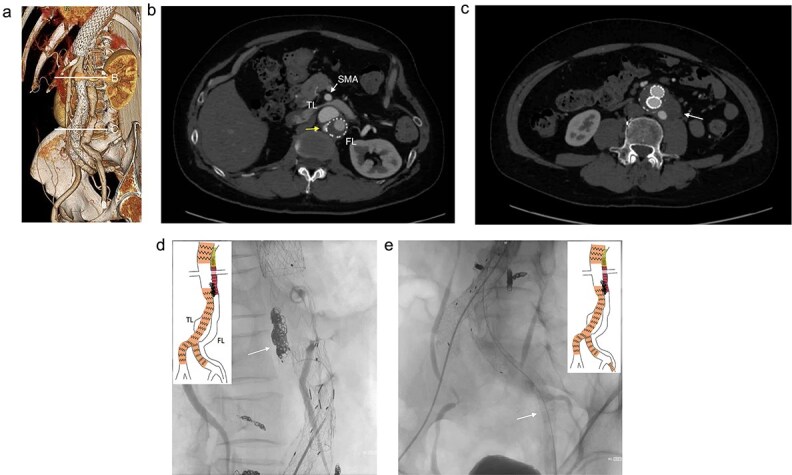
The contrasted CT 3D reconstruction 2 years after the false lumen (FL) stent graft placement (a; white arrow); the contrast-enhanced CT axial images showing small inflow to the FL from the true lumen through the gap between the intimal tear and the FL stent graft (b; arrow), and abdominal aortic aneurysm expansion to 56 mm (c; white arrow); the intraoperative angiography showing coiling of the gap between the intimal tear and FL stent graft (d; white arrow), and the VBX was deployed at left external iliac artery covering the re-entry tear (e; white arrow). TL: true lumen; FL: false lumen; SMA: superior mesenteric artery.

**Figure 3:**
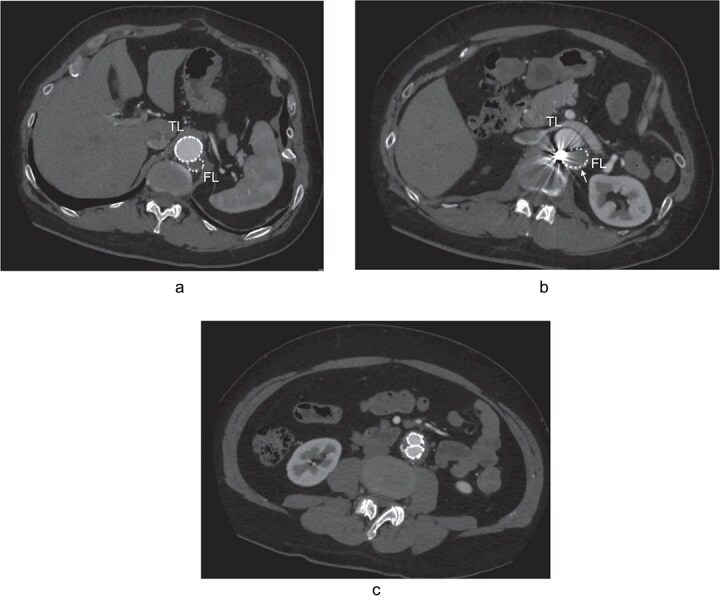
The postoperative contrast-enhanced CT axial images 3 years after the procedure showed no contrast inflow into the FL, and the reduced diameter of post-dissection thoracoabdominal aortic aneurysm at thoracic level (a), renal artery level with FL stent graft (b; white arrow), and abdominal level (c). TL: true lumen; FL: false lumen.

At 2-year post-procedure, CT follow-up showed AAA expansion to 56 mm, and FL dilation to 25 mm. The contrasted CT showed small inflow to the FL from the TL through the gap between the intimal flap and the FL stent graft via a residual intimal tear, as well as reversal flow from re-entry at the EIA (Fig. 2a–c). A second-stage endovascular repair was planned for completing the TAAA repair. Under local anesthesia, the gap between the FL stent graft and the intimal flap was occluded using DELTAFIL (Jonson & Jonson, NJ, USA) from FL side to close a residual intimal tear (Fig. 2d). The eighth intercostal artery and the third lumbar artery were occluded using DELTAFIL and GARAXY G3 (Jonson & Jonson, NJ, USA), respectively to block the backflow from these side branches. Then the 8 × 59 mm VBX (WL Gore & Associates, Newark, DE, USA) was deployed at left EIA covering the re-entry tear, successfully completing FL closure (Fig. 2e). The postoperative course was uneventful, with no paraplegia, and he was discharged on the postoperative day 4. The postoperative contrast-enhanced CT showed no contrast inflow into the FL. The CT imaging 3 years after the procedure showed the reduced diameter of abdominal aorta of 35 mm (Fig. 3a–c).

## Discussion

A previous study has demonstrated that FL stent graft placement successfully closed the intimal tears at the visceral segment, and could halt the dilation of the aneurysm associated with CTBAD [1]. At that time, the re-entry at the left EIA was left open intentionally, opting for a staged approach. This strategy preserved blood flow to several intercostal arteries originated from the FL, allowing time for collateral perfusion of the spinal cord to develop.

Open TAAA repair remains the gold standard for treating CTBAD, but it is highly invasive. Despite improved survival rates after TAAA repair, SCI remains a significant complication, with incidence rates ranging from 3% to 10%, even in highly experienced institutions [2]. Furthermore, in cases like this, where the lumbar artery originates from the FL, entry closure has been reported to carry an increased risk of SCI [3]. Therefore, staged repair has been reported as an effective method for reducing the risk of SCI during TAAA repair [4]. In the present case, staged endovascular closure of the intimal tears at the visceral segment, followed by closure of the re-entry tear at the EIA, enabled gradual reduction of FL blood flow. This approach helped preserve spinal cord perfusion and minimized the risk of SCI. After the first stage of treatment, it is crucial to conduct periodic CT follow-up, and if the TAAA shows signs of enlargement, the next intervention should be performed as promptly as possible.

FL stent graft placement represents a viable treatment option for CTBAD, though anatomical limitations exist. For example, while it is suitable when the visceral branches originate from the true lumen, it may not be feasible if these branches originated from the FL, as stent grafts could occlude the visceral branches. A previous study showed a case where the left renal artery, originating from the FL, was sacrificed during FL stent graft placement to treat a ruptured CTBAD as an emergent evacuation [5]. The FL stent graft can be a viable treatment option for patients at high risk for open surgery. However, the anatomical feasibility and the risks and benefits of FL stent graft placement should be carefully evaluated to determine whether the procedure is appropriate in each case.

FL stent graft placement is a valuable technique for sealing intimal tears at the visceral segment and can be an effective treatment option for post-dissection TAAA. A staged total endovascular repair approach, to close all intimal tears may lower the risk of SCI.

## Data Availability

Not applicable.
